# An improvement in acute wound healing in rats by the synergistic effect of photobiomodulation and arginine

**DOI:** 10.1186/s42826-019-0025-x

**Published:** 2019-12-11

**Authors:** Atarodsadat Mostafavinia, Mohammad Bidram, Amirhossein Gomi Avili, Mohammadamin Mahmanzar, Seyed Ali Karimifard, Ensieh Sajadi, Abdollah Amini, Mahsa Hadipour Jahromy, Seyed Kamran Ghoreishi, Sufan Chien, Mohammad Bayat

**Affiliations:** 1 Department of Anatomy, Faculty of Medicine, Tehran Medical Sciences, Islamic Azad University, Tehran, Iran; 2 Department of Microbiology, Faculty of Advanced Science and Technology, Tehran Medical Sciences, Islamic Azad University, Tehran, Iran; 3 Faculty of Medicine, Tehran Medical Sciences, Islamic Azad University, Tehran, Iran; 4 Department of Cellular and Molecular Biology, Faculty of Advanced Science and Technology, Tehran Medical Sciences, Islamic Azad University, Tehran, Iran; 5 Department of Biology and Anatomical sciences, School of Medicine, Shahid Beheshti University of Medical Sciences, Tehran, Iran; 6 Herbal pharmacology Research Center, Tehran Medical Sciences, Islamic Azad University, Tehran, Iran; 7 Department of Statistics, University of Qom, Qom, Iran; 8 Price Institute of Surgical Research, University of Louisville and Noveratech LLC of Louisville, Louisville, KY USA; 9 Department of Biology and Anatomical sciences, School of Medicine, Shahid Beheshti University of Medical Sciences, Tehran, Iran

**Keywords:** Acute wound healing, Arginine, Photobiomodulation, Tensiometrical properties, Stereology, Wound closure, Rat

## Abstract

In this probe, at first we examined the best route and dosage of arginine administration on wound healing in an excisional wound model in rats. Next, we intend to assess the impact of photobiomodulation (PBM) and arginine, individually and together, on the wound healing. In the pilot study, an excisional wound was made in each of 24 rats. There were 4 groups. Group 1 was the control group. In groups 2 and 3, wounds were topically treated with arginine ointments (ARG.) 2% and 5%, respectively. In group 4, arginine was injected (ARG. INJ.,i.p.). In the main phase, in 24 new rats, an excisional wound was made. There were 4 groups: group 5 served as the control. Wounds in group 6 were topically treated with ARG 2%. Wounds in group 7 were subjected to PBM. Wounds in group 8 were treated with PBM+ARG. 2%. On day 15, wound area measurement, wound strength, and stereological examination were performed. In the pilot study, we found that the ARG 2% ointment significantly decreased wound area than ARG. 5%, ARG. INJ. and control groups, and significantly increased wound strength compared to the control and ARG.5% groups. In the main phase, a significant decrease of wound area in all treatment regimens was induced. PBM + ARG. 2% and PBM treatment regimens significantly improved wound strength and almost all stereological parameters, compared to the control and ARG. 2% groups. PBM + ARG. 2% induced anti-inflammatory and angiogenic activities, and hastened the wound healing process in an excisional wound model in rats.

## Introduction

Globally, about 234 million vital surgeries are undertaken annually [1]. Worldwide assessments recommend that no less than 7 million patients suffer from side-effects after surgery yearly, consists of at least 1 million dying [2]. Approximately 50% of these deaths and complications including wound care matters, are avoidable [3].

Arginine is a basic amino acid that has many important functions in cell physiology. It is incorporated with protein production, but it is also closely incorporated with cellular signaling by synthesis of nitric oxide (NO) and cellular proliferation. Thus, arginine is a crucial substrate for skin injury repair course. Witte and Barbul [4]. Many probes have revealed that arginine supplementation could induce an increase of speed in curing. The outcomes from the earliest work by Shi et al. showed that a nutritional supplement of arginine improves skin injury repair in healthy mice [5]. In a rat model of skin injury repair, Witte et al. have evidenced that arginine supplementation recovers weakened curing in this skin injury animal simulation (model) with diabetes mellitus (DM) by regulating the NO path but then without disturbing arginase action [4]. Using a rat model, Shi et al. showed that the weakened curing following trauma or hemorrhage could be significantly improved by L-arginine supplementation [6]. Other researchers described that both arginine and fish oils alone have benefits, but the mixture seems to be considerably more effective. This mixture has been revealed to influence results concerning skin injury repair, and could also alter frequency and results in heart and vessels illnesses, DM, and other inflammatory diseases. However, above-mentioned potentials have not yet advanced to medical use globally [7]. Very recently, Durmus et al. proposed that topical use of a mixture of arginine, silicon, and inositol (ASI) cream has favorable impacts on the curing reaction of an excisional skin injury simulation in rats [8]. Further documents for beneficial effectiveness of arginine in skin injury repair in sick persons originates from a probe by Debats et al. who described that arginine supplementation hastens superficial skin injuries in sick persons [9]. In another study Liu et al. have shown that supplementation with high-arginine foods directed to an important enhancement in pressure ulcer (PU) curing [10]. In spite of the above-mentioned inspiring outcomes in animal simulations, none of the available clinical trials has revealed efficiency of L-arginine at dosages beyond average nutritional practices on the result in unfavorably ill surgical patients, as well as decrease in infectious difficulties [11]. Additionally, the optimum mixtures of immunonutrients, scheduling of management, and the dosages required for finest outcomes need to be resolved in animal studies and clinical probes [7].

Photobiomodulation (PBM) is the use of laser to trigger curing and decrease inflammation [12]. Gal et al. in his review article stated that PBM is capable to boost collagen production, wound strength and to increase wound closure in animals [13]. On the other hand Reddy, and Beckmann et al. in their review articles stated that the majority of animal and clinical probes display a probable advantage of PBM in skin injury repair of healthy and diabetic wounds [14, 15]. Nevertheless, there are many facts in these probes that alter concluding documents about the real output of PBM [14]. In brief, all the above-mentioned probes provide sufficient documents to keep investigating PBM for diabetic wounds, but human studies do not offer sufficient evidence to confirm the worth of PBM as a persuasive modality in wound care management currently. Additional well-planned investigations and clinical trials are essential to conclude the true worth of PBM in current wound management [14].

Many factors could affect one or more steps of skin injury repair course, therefore producing improper or impaired skin injury repair [16]. Arginine is the nitrogenous predecessor for NO production and it regulates vital metabolic pathways [17]. PBM decreases inflammation, and enhances repair, and inhibits cell and tissue impairment [18]. In some studies, the favorable application of a mixture of remedial modalities and medicines have been exposed, since in animal simulations of skin injuries and ulcers they validate a synergistic effect and could improve the success of cure in different skin injuries and ulcers [7, 19, 20]. We hypothesize that a mixture of beneficial biomediators (PBM and arginine) could be applied to hasten the curing in skin injury because of their anti-inflammatory activities and triggering effects in cellular physiology. In this study, initially, we examined the best route and dosage of arginine administration on skin injury repair in an excisional wound simulation. Next, we intend to assess the effect of PBM and arginine, individually and together, on the wound closure, wound strength, and stereological parameters in a rat simulation of excisional skin injury simulation.

## Materials and methods

### Study design and animals

The clinical ethics office at Shahid Beheshti University of Medical Sciences (SBMU), permitted this probe (IR.SBMU.MSP.REC.1398.107). In order to achieve our investigation aims our probe is allocated in two different phases as explained below. In the first phase, we want to define whether route and dosage of arginine administration could significantly hasten skin injury repair of rats. In the next phase, we will evaluate the influence of PBM and arginine administration individually and in combination on skin injury repair. At first phase, 24 male Wistar rats were used and a skin injury was made in each rat. All rats were arbitrarily distributed into four groups of six rats per group as follows: group one served as control and their wounds did not receive any treatment. In groups 2 and 3, wounds were topically treated with arginine ointment (ARG.) 2 and 5% respectively. In group 4 (ARG. INJ.), 200 mg/kg of arginine was intraperitoneally injected. Each treatment was performed once a day. On day 15, wound closure, and wound strength examinations were performed. In the next phase, another 24 male Wistar rats were used and a skin injury was made in each rat. All rats were arbitrarily distributed into 4 groups of six rats per group as follows: group 5 served as the control group. Wounds in group 6 were topically treated with ARG 2%. Wounds in group 7 (PBM) were subjected to PBM. Wounds in group 8 were treated with PBM+ ARG 2%. On day 15, wound closure, wound strength, and stereological examinations were performed. All wounds were observed every day and body weight of rats were recorded during the study.

### Phase one, pilot for determining best arginine administration method

#### Surgery for induction of skin injury

**Fig. 1:**
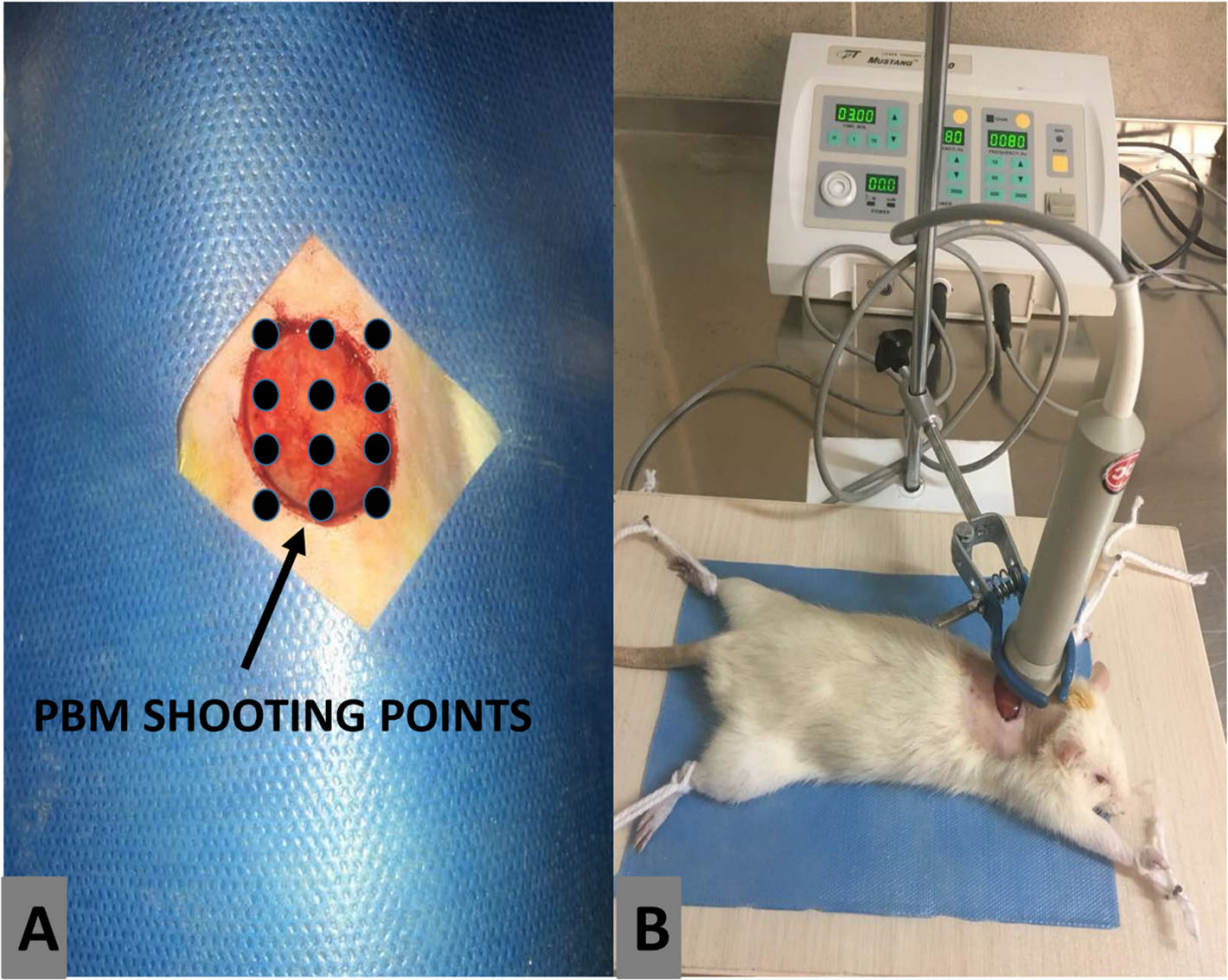
A photo of wound, and photobiomodulation target sites

The rats were anesthetized by ketamine (50 mg/kg, i.m.) and xylazine (5 mg/kg, i.m.). The surgery was performed under aseptic conditions. One 15 mm full thickness excisional round skin injury that included the skin muscle was produced in the proximal part of the back of rats by a scalpel no. 15 (Fig. 1). The day of surgery was named as day zero.

#### Preparation and administration of arginine

We dissolved 2 g and 5 g of arginine powder (Sigma-Aldrich, USA) in 2 jars of water and added 98 g and 95 g of Eucerin under sterile conditions to create the arginine ointments with 2 and 5% densities, respectively. Approximately 0.04 g of the arginine ointments were topically applied to the wounds of groups 2 and 3 once a day. For arginine injection, at first, 0.2 g of arginine powder was dissolved in 100 cc distilled water under sterile conditions. Next, 20 mg/kg of the solution was injected intraperitoneally once a day to the rats of group 4.

#### Wound area measurement method

We used a digital camera to take pictures of the skin injuries on day 15. The skin injury areas were calculated by image J-NIH (USA). Skin injury area measurement was used to show and measure wound closure [21].

#### Wound strength examination

One uniform sample (5 × 50 mm band) was taken from each skin injury of the euthanized rats at day 15, and fixed in a material testing device. The deformation ratio was 10 mm/min. We used the load-deformation curve to calculate the bending stiffness (M Pa), maximum force (N), stress high load (N/cm^2^), and energy absorption of the samples (J) [20, 22].

#### Statistical analysis

Data are presented as mean ± standard deviation (SD). We used the t-test, one-way analysis of variance (ANOVA), and the least significant difference (LSD) tests for statistical analyses. A *p*-value of < 0.05 was identified significant.

#### Phase 2, main part

Twenty-four male Wistar rats were used and a skin injury was made in each rat. All rats were arbitrarily distributed into four groups of six rats per group as follows: group 5 served as the control group. Skin injuries in group 6 were topically treated with approximately 0.04 g of the ARG 2%. Skin injuries in group 7 were subjected with PBM. Skin injuries in group 8 were treated with PBM+ arginine ointment2%. On day 15, wound closure, wound strength, and stereological examinations were performed.

#### PBM

The skin injuries in the PBM-treated rats were subjected to a laser [MUSTANG 2000 with LO7 probe; Technica Co., Russia], using the following specifications:

Power density: 1.08 mW/cm^2^, Peak power output: 75 W, Spot size: 1 cm^2^, Pulse rate: 80 Hz, Wavelength: 890 nm, Pulsed duration: 180 ns, Energy density: 0.2 J/cm^2^, Duration of shooting for each point: 200 s., Number of PBM shootings in each session: 12. PBM was started immediately after surgery and continued once daily, 6 days a week for 15 days (Fig. 1).

#### Stereological examination

On day 15 a sample from the skin injury was excised, and prepared for light histological study, and 10 serially sectioned slides were stained with Hematoxylin & Eosin method. The physical dissector technique was used to calculate the numerical density (Nv) of neutrophils, fibroblasts, length density of the vessels, and basal cells numbers of new epidermidis [20]:

$$ \mathbf{Nv}=\left[\varSigma Q\;\left( number\ of\ nuclei\right)/h\left( the\ height\ of\ the\ dissector\right)\times a/f\left( counting\ frame\ area\right)\times \varSigma p\;\left( number\ of\ counting\ frames\ in\  all\  fields\right)\right]\times \left[t\;\left( real\ section\ thickness\right)/ BA\ \left( section\kern0.17em thickness\right)\right] $$

$$ \mathbf{N}(total)= Nv\ \left( numerical\ density\right)\times V\ \left( total\ volume, final\right) $$

#### Estimation of the length density of the vessels (lv)

Lv, was considered as a stereological marker for new blood formation) [20]:

$$ Lv=2\varSigma Q\ \left( total\ number\ of\ the\ vessel\ profiles\ counted\  per\  rat\  skin\right)/\Big(\varSigma P\ \left( number\ of\ counting\ frames\ in\  all\  fields\ \left(a/f\right)\right) $$

#### Estimation of the volume of new epidermidis, and new dermis

The total volume (V, mm^3^) of new epidermidis, and new dermis was calculated by the Cavalieri’s method [23]:

$$ V=\varSigma P\ \left( total\ number\ of\ the\ volume\ profiles\ counted\  per\  rat\hbox{'} skin\right)\times a/p\ \left( the\ area\ interrelated\ to\ each\ specific\ point\ projected\  on\  the\ tissue\right)\times t\ \left( the\ distance\ between\ the\ sampled\ sections\ perceivably\right) $$

It is noted that surgery for induction of skin injury, wound area measurement method, wound strength examination, and statistical analysis sections of this phase were the same as phase one.

## Results

### Clinical observations

There were not any skin injury exudate or other clinical complications during the probe. There were significant changes in the rats’ weights of some study groups on day 15, compared to day zero (Table 1).

**Table 1:** Mean ± Standard Deviation of body weight changes of studied groups

PHASE	GROUPS	PRIMARY WEIGHT	SECONDARY WEIGHT
1, PILOT	CONTROL	254.8 ± 25.4	226.0 ± 25.4***
1,PILOT	ARG. 2%	274.0 ± 17.1	271.5 ± 13.98
1, PILOT	ARG. 5%	333.7 ± 22.0	257.2 ± 35.9**
1, PILOT	ARG. INJ.	335.2 ± 38.4	295.2 ± 36.9***
2, MAIN PART	CONTROL	255.0 ± 26.0	2256.7 ± 26.0***
2, MAIN PART	ARG. 2%	273.0 ± 17.0	270.5 ± 14.0
2, MAIN PART	PBM	278.2 ± 34.40	294.50 ± 16.5**
2, MAIN PART	PBM + ARG. 2%	251.00 ± 57.24	225.25 ± 57.30***

*ARG* arginine, *INJ* injection, *PBM* photobiomodulation; ** < 0.01, *** < 0.001

### Phase 1, wound area measurement findings

**Fig. 2:**
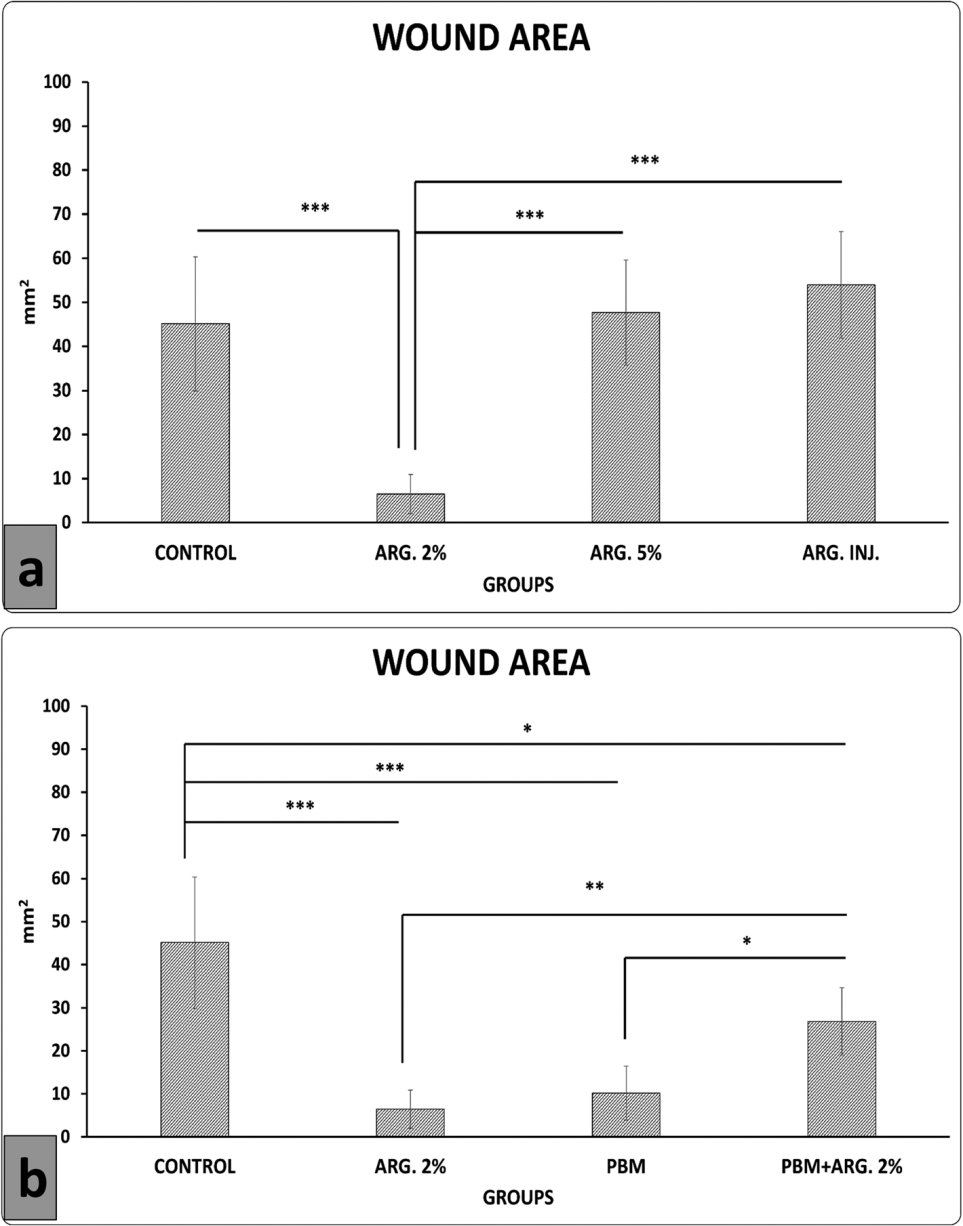
Mean ± standard deviation (SD) of the wound area measurement in the pilot study phase (**a**), and main phase (**b**), as compared by analysis of variance (ANOVA) and least significant difference (LSD). *p<0.05, **p<0.01, and ***p<0.001. *ARG* arginine; *INJ* injection; *PBM* photobiomodulation

Stereological results were shown in Additional file 1 named stereological findings of phase one. All *p*-values were related to LSD test. Figure 2a shows that ARG. 2% treatment significantly decreased wound area compared to other groups (all *p* = 0.000).

### Phase 2, wound area measurement findings

Figure 2b shows that ARG. 2%, PBM and PBM + ARG. 2% treatments significantly decreased wound area than the control group (*p* = 0.015, *p* = 0.000, *p* = 0.000). Concurrently, ARG. 2% and PBM treatments were significantly more effective than PBM+ ARG. 2% treatment (*p* = 0.008, *p* = 0.025).

### Phase 1, wound strength results, bending stiffness (M pa)

**Fig. 3:**
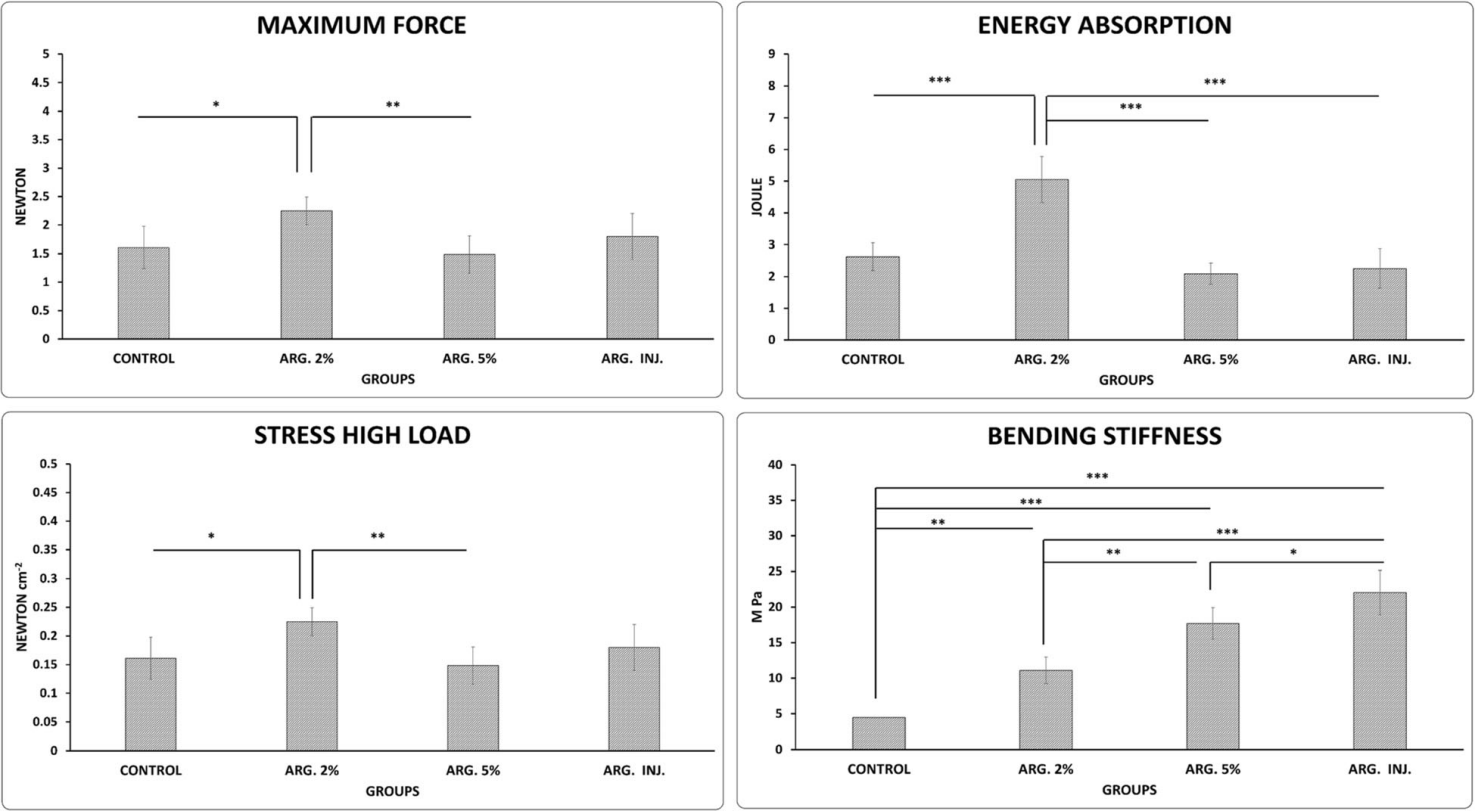
Mean ± SD of the bending stiffness, maximum force, stress high load, and energy absorption in the pilot study phase, as compared by ANOVA and LSD, *p<0.05, **p<0.01, ***p<0.001

Figure 3a shows that ARG. INJ., ARG. 5%, and ARG. 2% treatments significantly increased bending stiffness compared to the control group (*p* = 0.000, *p* = 0.000, *p* = 0.221). At the same time, ARG. INJ. and ARG. 5%, were statistically better than ARG. 2% (*p* = 0.000, *p* = 0.001). And ARG. INJ. was statistically better than ARG. 5% (*p* = 0.014).

### Maximum force (N)

Figure 3b shows that ARG. 2% treatments significantly increased maximum force compared to ARG. 5% and the control groups (*p* = 0.008, *p* = 0.021).

### Stress high load (N/cm^2^)

Figure 3c shows that ARG. 2% treatments significantly increased stress high load compared to ARG. 5% and the control groups (*p* = 0.008, *p* = 0.021).

### Energy absorption (J)

Figure 3d shows that ARG. 2% treatments significantly increased energy absorption than other groups (all *p* = 0.000).

### Phase 2, wound strength results, bending stiffness (M pa)

**Fig. 4:**
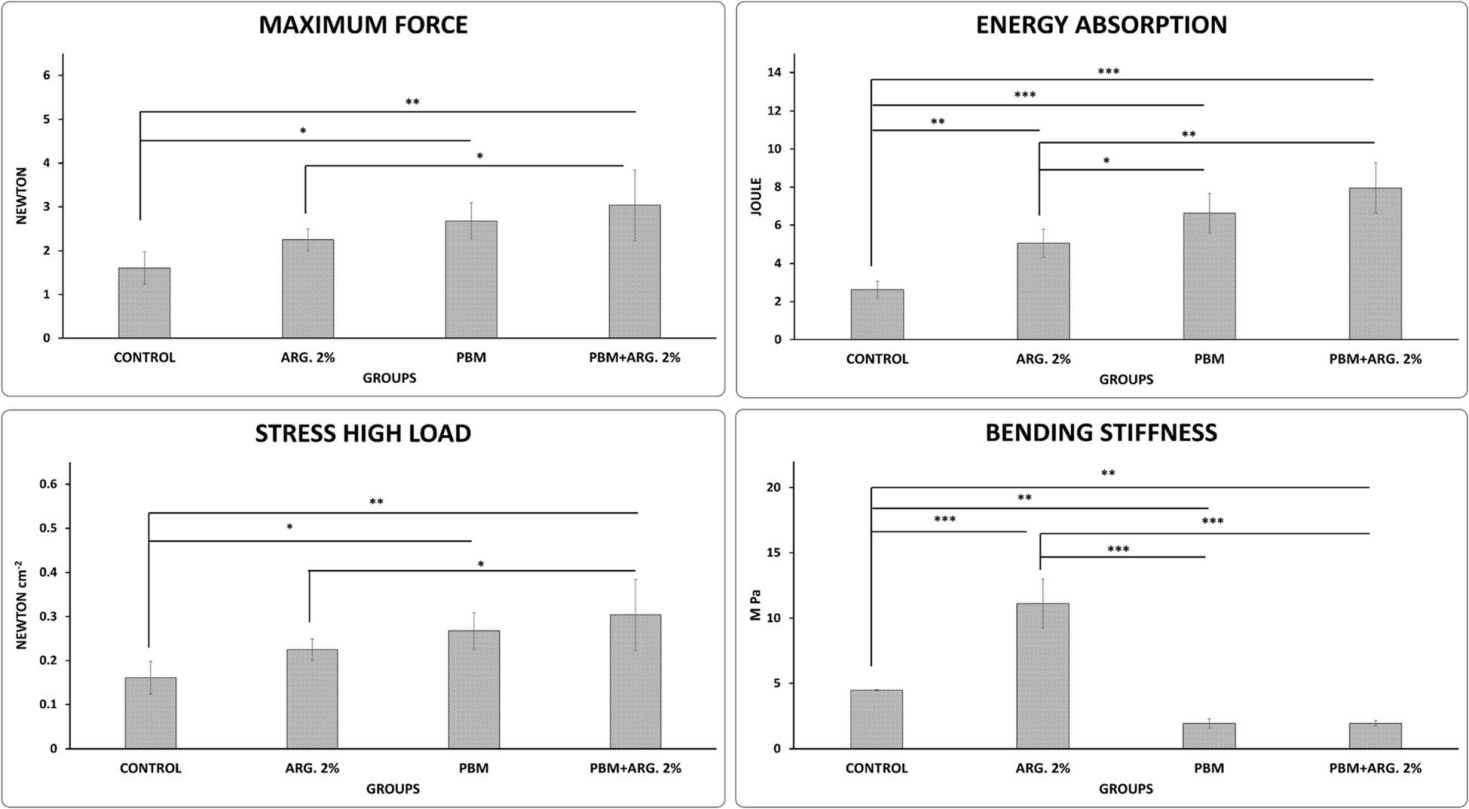
Mean ± SD of the bending stiffness, maximum force, stress high load, and energy absorption in the main phase, as compared by ANOVA and LSD, *p<0.05, **p<0.01, ***p<0.001

Figure 4a shows that ARG. 2% treatment significantly increased bending stiffness than other groups (all *p* = 0.000). Concurrently, PBM and PBM + ARG. 2% treatments were statistically inferior to the control group (*p* = 0.002. *p* = 0.001).

### Maximum force

Figure 4b displays that PBM + ARG. 2% treatment significantly increased maximum force compared to the control and ARG. 2% groups (*p* = 0.002, *p* = 0.015). Concurrently, PBM + ARG. 2% treatment was statistically better than ARG. 2% (*p* = 0.046).

### Stress high load (N/cm^2^)

Figure 4c indicates that PBM + ARG. 2% treatment significantly increased stress high load compared to the control and ARG. 2% groups (*p* = 0.002, *p* = 0.015). Concurrently, PBM + ARG. 2% treatment were statistically better than ARG. 2% (*p* = 0.046).

### Energy absorption (J)

Figure 4d shows that PBM + ARG. 2%, PBM, and ARG. 2% treatments significantly increased energy absorption than the control group (*p* = 0.000, *p* = 0.000, and *p* = 0.004). At the same time, PBM + ARG. 2% and PBM treatments were statistically better than ARG. 2% (*p* = 0.001, *p* = 0.041).

### Phase 2, stereological findings

**Fig. 5:**
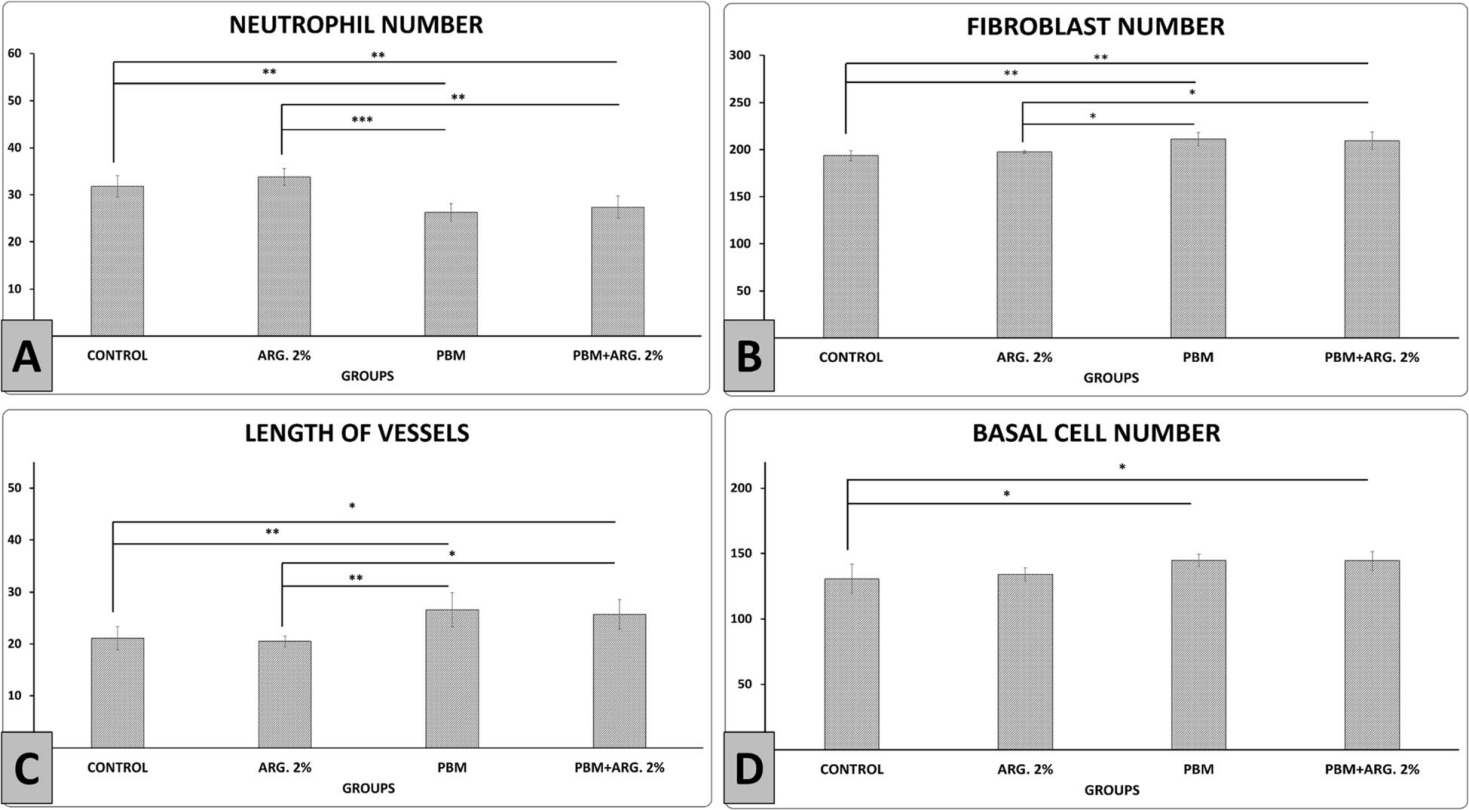
Mean ± SD of the neutrophil, fibroblast, angiogenesis, and basal cell number of new epidermidis in the main phase, as compared by ANOVA and LSD, *p<0.05, **p<0.01, and ***p<0.001. *ARG* arginine; *INJ* injection; *PBM* photobiomodulation

Figure 5a shows PBM+ ARG. 2%, and PBM treatments significantly decreased neutrophils number than the control (*p* = 0.006, *p* = 0.002) and ARG. 2% treatment (*p* = 0.001, *p* = 0.000). Figure 5b shows that PBM+ ARG. 2%, and PBM treatments significantly increased fibroblasts number compared to the control group (*p* = 0.006, *p* = 0.005). Concurrently, PBM treatment was statistically better than ARG.2% treatment (*p* = 0.036). Figure 5c shows PBM+ ARG. 2%, and PBM treatments significantly increased new blood vessel formation compared to the control group (*p* = 0.014,*p* = 0.008) and ARG. 2% (*p* = 0.001, *p* = 0.000). Figure 5d shows that PBM+ ARG. 2% and PBM treatments significantly increased basal cell number of new epidermidis compared to the control group (*p* = 0.015,*p* = 0.019).

**Fig. 6:**
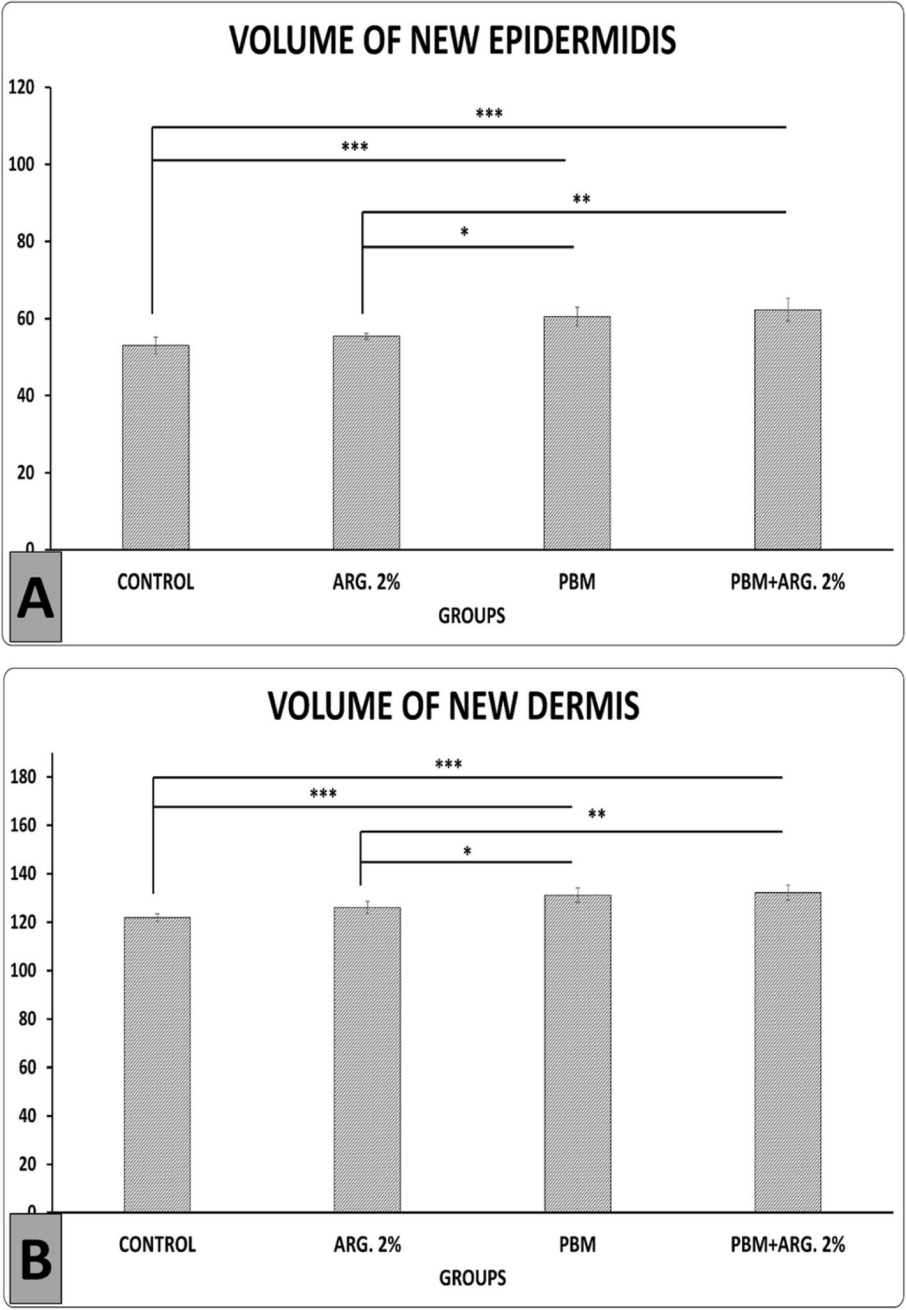
Mean ± SD of the volume of new epidermidis and volume of new dermis in the main phase, as compared by ANOVA and LSD tests, *p<0.05, **p<0.01, ***p<0.001

Figure 6a shows PBM+ ARG. 2%, and PBM treatments significantly increased volume of new epidermidis in comparison with the control group (LSD test, both *p* = 0.000) and ARG. 2% treatment (*p* = 0.002, *p* = 0.014). Figure 6b shows that PBM+ ARG. 2%, and PBM treatments significantly increased the volume of the new dermis in comparison with the control group (both *p* = 0.000) and ARG. 2% treatment (*p* = 0.007, *p* = 0.025).

## Discussion

L-Arginine is in the category of safe and nutritional supplements, and so far, the use of its ointment on wound healing has not been reported. Also 2 and 5% skin ointments are common in practice and in the market [24]. There is a 2.5% L-Arginine ointment which is used to increase blood flow in the market. Therefore, for pilot, and for the first time, we used two doses 2 and 5% of arginine for topical usage on wound. There was also one group with injection form. Since arginine 5% and injection form did not yield the desired results, we excluded them in the second part of the study. Using two different routes of administration, two different doses of arginine, and PBM, are the innovations of this study. In addition for the first time we suggest a 2% of arginine ointment for treating topically wounds [4].

It should be noted that all rats had the same sexuality, and race, and were in the same nutrition and maintenance conditions but the response of them to stressors (wounds, PBM, intentional and topical forms of arginine) were different. Although the rats lost weight in the PBM + Arg.2% group, their wound healing was better than the other groups. Moreover, the PBM group had only more energy absorption level than the control group despite weight gain. In the first phase of the study, although arginine 2% caused to reduce the weights in rats, the wound healing was better in this group than the others were. It seems that the rats had different reactions to the nutrition and the stressor items including wound, various arginine doses, PBM and their combination effects.

Herewith, we hypothesize that a mixture of PBM and arginine could quicken the skin injury repair course because of their anti-inflammatory activities and triggering effects respectively. In the pilot study, we found that ARG. 2% treatment significantly decreased wound area compared to ARG. 5%, ARG. INJ. and control groups, and significantly increased wound strength compared to control and ARG.5%. Thus, we selected ARG. 2% for the main phase of our study. In the main phase, we found that all treatment regimens significantly decreased wound area. But PBM and ARG. 2% were statistically better than PBM + ARG. 2%. In the case of wound strength and stereological examinations, PBM + ARG. 2% and PBM regimens significantly improved wound strength and almost all stereological parameters in comparison with the control and ARG. 2% groups [4].

Skin injury repair displays a series of actions categorized by the reorganization of the injured skin in an attempt to re-establish the integrity of the damaged skin. The course include noticeably complicated factors involving the production of the matrix constituents and collaborations among many factors such as growth factors [25]. Investigation for factors influencing and hastening skin injury repair is quickly in advancement. Since skin injury repair is a well-coordinated course with many factors, a simple stimulation of one molecule may not result in effectiveness of positive curing [26]. Thus, in the present probe, we have assessed the influence of PBM and ARG. 2% treatments alone or together on an excisional skin injury repair simulation in rats, in order to find a well-curing outcome. Wound contraction is the chief aim of wound treatment. After surgery, wound reopening is a frequent obstacle in surgical procedures. The severity of wound reopening might exhibit from minor conditions that need local wound care to severe conditions with recurrent surgical interventions and a high death ratio [27]. In this study, all treatment regimens significantly hastened wound closure as compared to the control group on day 15. According to the wound area measurements, the skin injury repair was observed to be statistically better in PBM, and ARG. 2% treatments.

Probes have revealed that L-arginine, by its multipurpose metabolic and functional paths, could advance many cellular actions. To summarize some of its impacts; L-arginine is contributed in the synthesis of many enzymes, hormones, and structural proteins. It is the functional predecessor of different important biological molecules such as NO and polyamines. As a booster for the immune system, arginine triggers the thymus and stimulates lymphocyte production. This might be a vital key for arginine’s capability to stimulate repair of burns and other skin injuries [28].

Hence, in recent years, noteworthy consideration has been given to take arginine to the clinical practice for enhancing of skin injury repair, such as many of the human studies [9, 29], and animal study probes [5, 6].

However, improvements in the skin injury repair issue and the medicinal use of arginine have still been delayed because of its antiproliferative feature at high doses [30]. unfavorable gastrointestinal impacts [31], some adverse complications and fast presentation of drug tolerance [32], and weakened insulin sensitivity [33]. Accordingly, Loehe et al. stated that regardless of some favorable outcomes of arginine in animal simulations, none of the available human studies have confirmed the effectiveness of L-arginine at concentrations beyond regular nutritional practices on the result in critically-ill surgical patients, excluding the decrease in infectious difficulties [11]. The tensiometerical parameters of repairing skin injuries are important because they identify dehiscence of the repairing skin injuries. Sometimes, medical difficulties in diabetic ulcers of patients arise as the repaired ulcers frequently reopen, even with the least movement. When repaired skin injuries reopen, they need additional attention [34].

In the current probe, as stated by the wound strength examination, the skin injury repair was detected to be significantly better in PBM + ARG. 2% and PBM treatments than ARG. 2% and control groups. PBM + ARG. 2% and PBM treatments (especially PBM + ARG. 2% treatment) also significantly improved almost all studied stereological parameters of new skin injury bed and new epidermidis. It could be interpreted that the arginine bioavailability was higher under influence of the PBM treatment.

Escalation in arginine bioavailability causes vasodilatation of vasculatures, to permit for ideal blood flow by vasculatures, and raises blood supply to the skin injury. It also could suggest the means to reduce blood vessels dysfunction and also reduces the pathophysiology related to the skin injury repair. NO might increase with the escalation in arginine bioavailability leading to vasculature to expand, and increased blood supply to the place of skin injury [35]. In addition, arginine is a predecessor to proline, which is changed to hydroxyproline and next to collagen which would promote curing of damaged skin [36]. Our results are consistent with those of previous reports. Alexander and Supp presented that both arginine and fish oils are good individually but the mixture seems to be much more persuasive. This mixture has been demonstrated to alter outcomes of skin injury repair and infections, and could also alter incidence and outcomes in heart and blood vessel diseases, DM, and other inflammatory diseases [7]. In Durmus et al. study, the skin injuries of the experimental groups were treated by 4 or 10% ASI cream two times a day. The animals were euthanized either 5, 10, or 15 days after surgery, and samples were extracted for biochemical, molecular and histological examinations. Durmus et al. found that granulation tissue appeared meaningfully quicker in the ASI-treated groups compared to the control groups. Durmus et al. also measured some important molecules in new skin injury bed. Durmus et al. observed a significant increase in expression level of the molecules in ASI-treated skin injuries. Durmus et al. concluded that topical use of ASI cream (particularly 4% density) has favorable impacts on the curing reaction of an excisional skin injury simulation in rats [8]. Angiogenesis plays an important function in skin injury repair [10]. PBM + ARG. 2% and PBM treatments had evidence of reduced neutrophils and increased the length density of the vessels, which supported the anti-inflammatory and repair role of PBM + ARG. 2%, and PBM treatments in skin injuries of rats. Overall, our findings supported the outcomes reported by Fridoni et al. and Amini et al. Fridoni et al. examined the impacts of PBM (890 nm, 80 Hz, 0.2 J/cm^2^, once a day) and conditioned medium (CM, 4 times injections) of mesenchymal stem cells (MSC) alone or together on the histomorphological parameters in an infected skin injury in streptozotocin (STZ)-induced type-1 DM simulation in rats. Fridoni et al. determined that the use of PBM + CM induced anti-inflammatory and angiogenic activities, and hastened skin injury repair [37]. Amini et al. explored the impacts of PBM (890 nm, 80 Hz, 0.2 J/cm^2^, once a day) individually and together with curcumin on histomorphological parameters in a secondary intention skin injury in a STZ-induced type 1 DM simulation in rats. Amini et al. found that the PBM and PBM + curcumin groups had significantly better inflammatory reaction in comparison with the other groups at days 4, 7, and 15 after surgery. Amini et al. found that both the PBM and PBM + curcumin groups meaningfully improved skin injury repair by modification of the inflammatory cells count. Amini et al. determined that the PBM and PBM + curcumin treatments meaningfully improved the skin injury repair course to more quickly attain the proliferation step of the skin injury repair in rats with type-1 DM [20]. Amini et al. in another probe examined the impacts of CM from MSCs (two times administrations) and PBM (890 nm, 80 Hz, 0.2 J/cm^2^, once a day), applied alone or together, on the histomorphological parameters and gene expression of some related cytokines in a secondary intention skin injuries of STZ-induced of type-1 DM simulation in rats. In Amini et al. probe, the histomorphological parameters of the proximal and distal skin injuries exposed meaningfully boosted curing in all of the experimental groups, in comparison with the control group. The degree of curing was meaningfully superior in the CM + PBM group than in the other group. Amini et al. concluded that application of CM and PBM, alone or together, hastened the course of skin injury repair in rats with type-1 DM. The outcomes of CM + PBM group showed a synergistic impact, and it was meaningfully more effective than alone uses of CM or PBM [20].

In current probe, we observed significant decreased of the number of basal (epidermal) cells, fibroblasts and decreased of epidermal and dermal volumes, in the arginine 2% group (Figs. 5 and 6). These changes indicate that this group has entered the remodeling phase of wound healing earlier than other groups.

Figure 2a showed that there was a significant decrease in wound area in ARG 2% relative to ARG 5%.

So it was concluded that the effect of arginine is dose-dependent, and a lower dose significantly stimulates the cellular and molecular pathways, thereby accelerates wound healing.

## Conclusions

In the pilot study, ARG. 2% treatment significantly hastened excisional skin injury repair than ARG. 5%, and the control groups. Thus the effect of arginine is dose-dependent. In the main phase all treatment regimens significantly boosted wound closure of an excisional skin injury simulation in healthy rats. In cases of wound strength and stereological examinations, PBM + ARG. 2% and PBM treatments boosted wound strength and improved almost all stereological parameters. Thus, PBM + ARG. 2% prompted anti-inflammatory and angiogenic activities, and hastened skin injury repair course in an excisional skin injury simulation in rats. We suggest additional preclinical studies assess the uses of PBM with PBM + ARG. 2% treatments to treat diabetic ulcers and infected skin injuries of animal simulation in a try to reduce skin injury complications and promote repair.

## Supplementary information

**Additional file 1.** Stereological findings for phase one.

## Data Availability

There was some supporting data available for this work. The datasets used and/or analyzed in this study are available from the corresponding author on reasonable request.

## Abbreviations

ANOVA: Analysis of variance
ARG: Arginine ointment
ASI: Arginine, silicon, and inositol
CM: Conditioned medium
DM: Diabetes mellitus
INJ: Injection
LSD: Least significant difference
MSC: Mesenchymal stem cells
NO: Nitric oxide
Nv: Numerical density
PBM: Photobiomodulation
PU: Pressure ulcer
SBMU: Shahid Beheshti University of Medical Sciences
SD: Standard deviation
STZ: Streptozotocin
