# Supplementary material for: An improvement in acute wound healing in rats by the synergistic effect of photobiomodulation and arginine
Source: Lab Anim Res. 2019 Dec 11;35:28. doi: 10.1186/s42826-019-0025-x (PMC7081532; doi:10.1186/s42826-019-0025-x)
Supplement: Supplementary file 1 — Additional file 1. Stereological findings for phase one. [file 42826_2019_25_MOESM1_ESM.zip › Stereological findings of phase one or (Streological results of phase one).pptx]

## Slide 1
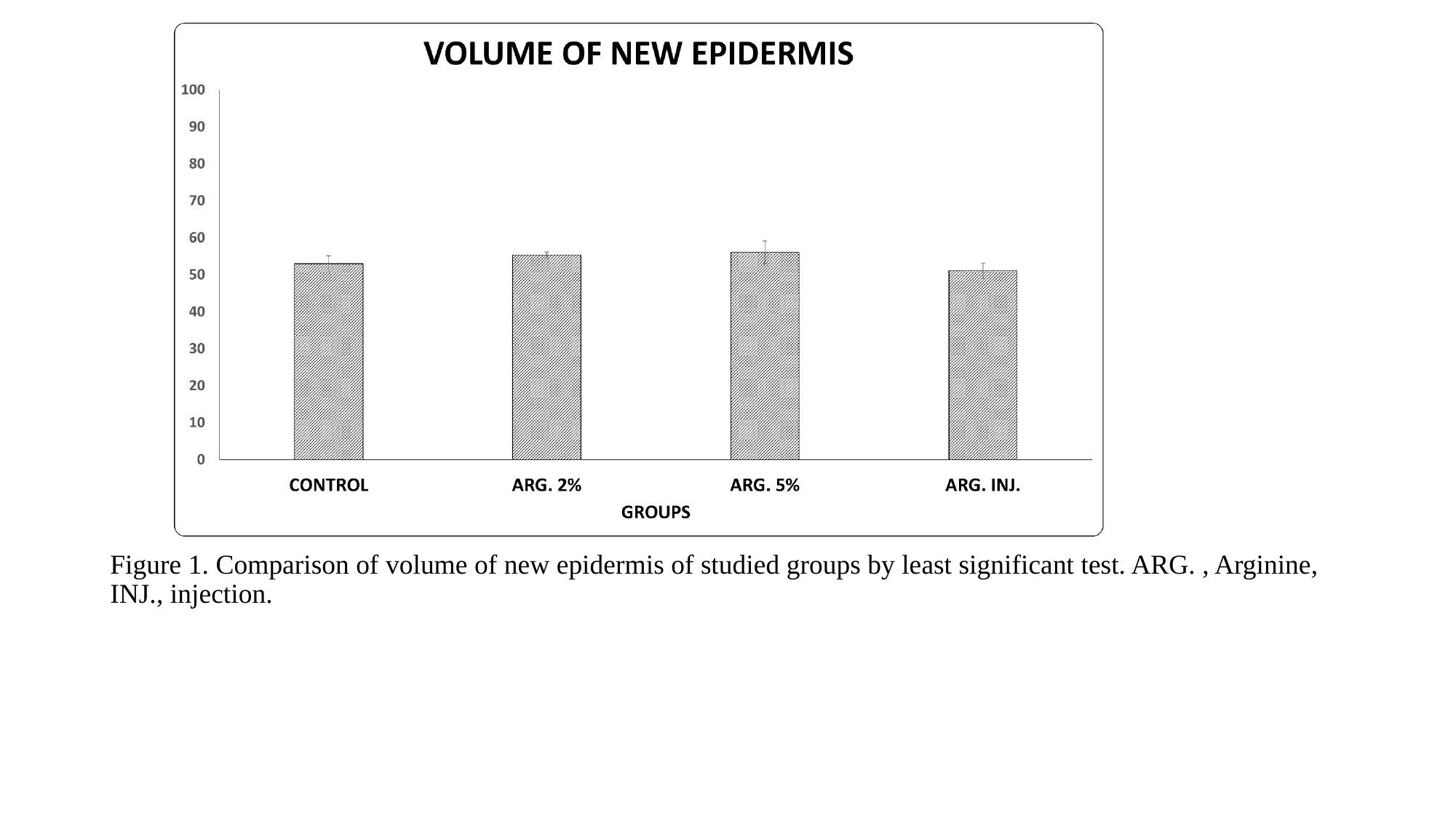

Figure 1. Comparison of volume of new epidermis of studied groups by least significant test. ARG. , Arginine, INJ., injection.

## Slide 2
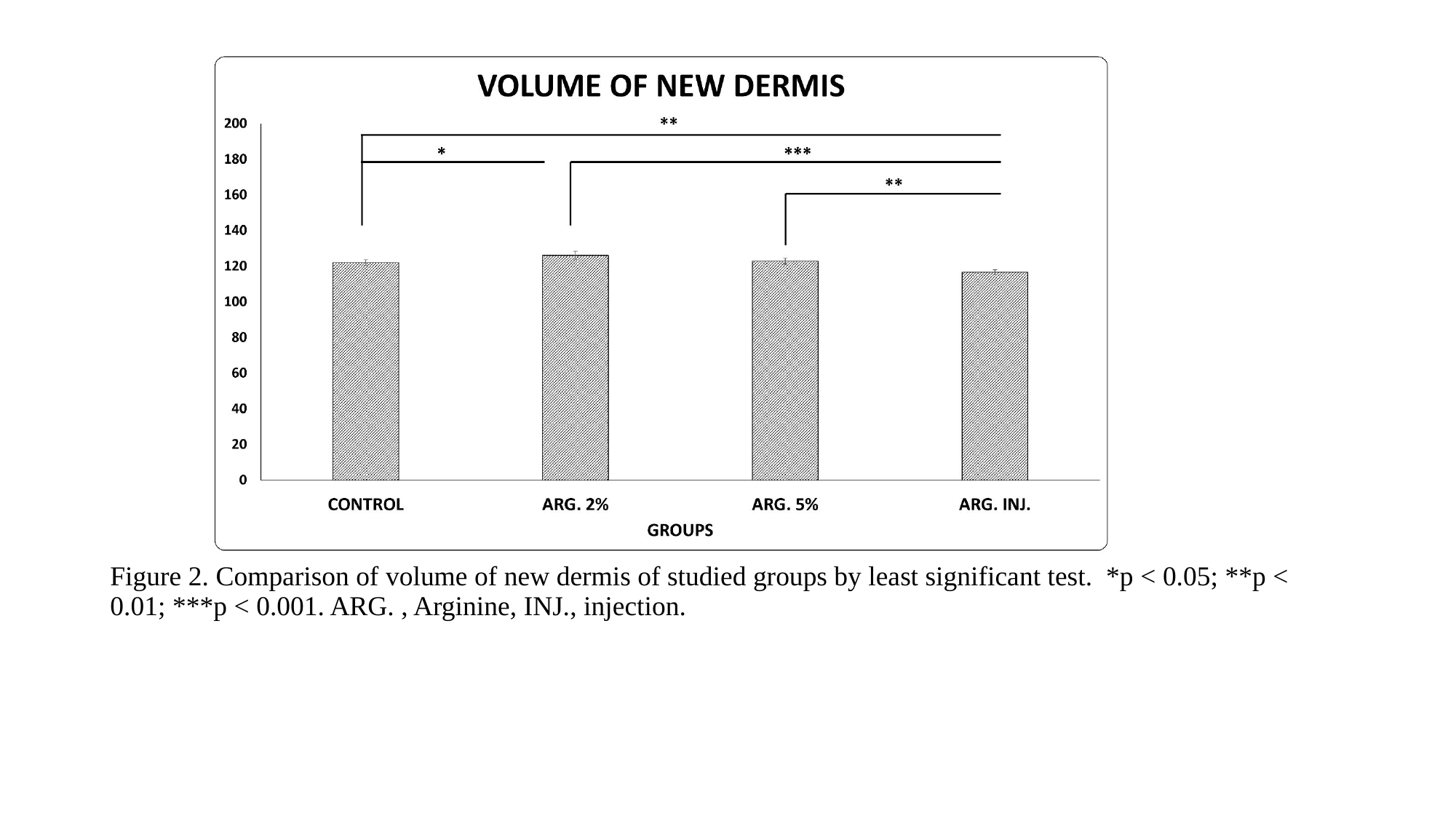

Figure 2. Comparison of volume of new dermis of studied groups by least significant test. *p < 0.05; **p < 0.01; ***p < 0.001. ARG. , Arginine, INJ., injection.

## Slide 3
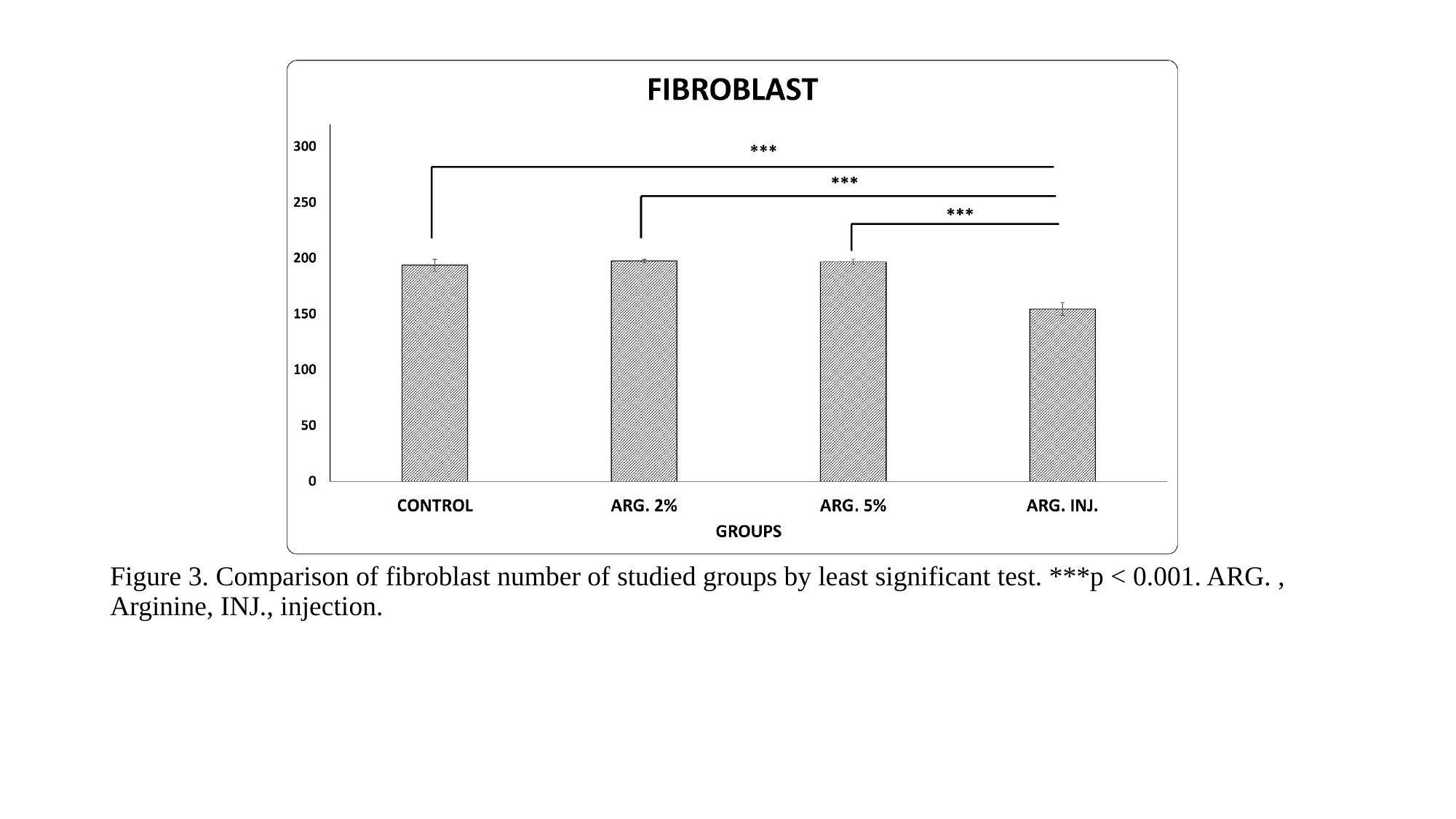

Figure 3. Comparison of fibroblast number of studied groups by least significant test. ***p < 0.001. ARG. , Arginine, INJ., injection.

## Slide 4
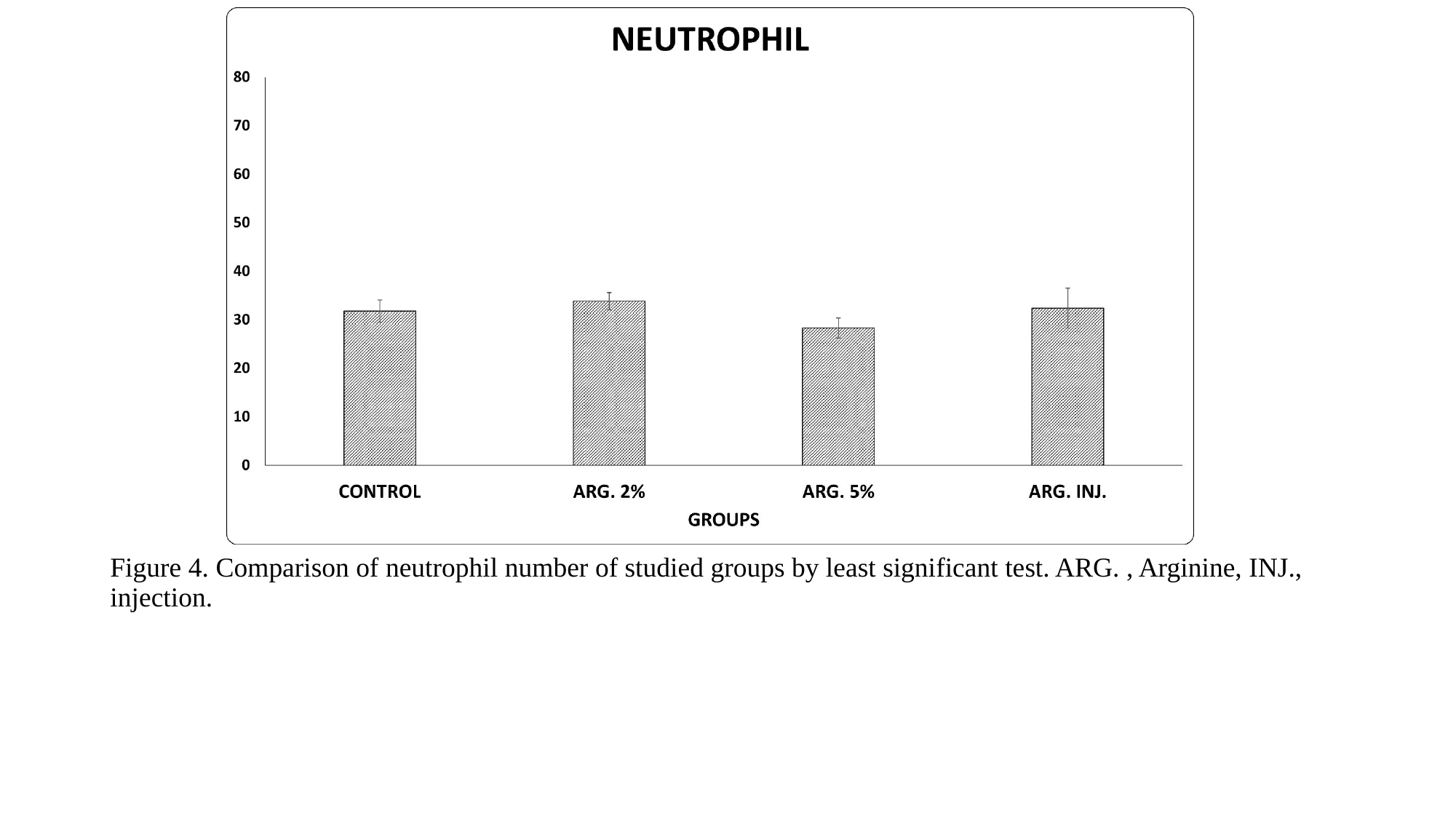

Figure 4. Comparison of neutrophil number of studied groups by least significant test. ARG. , Arginine, INJ., injection.

## Slide 5
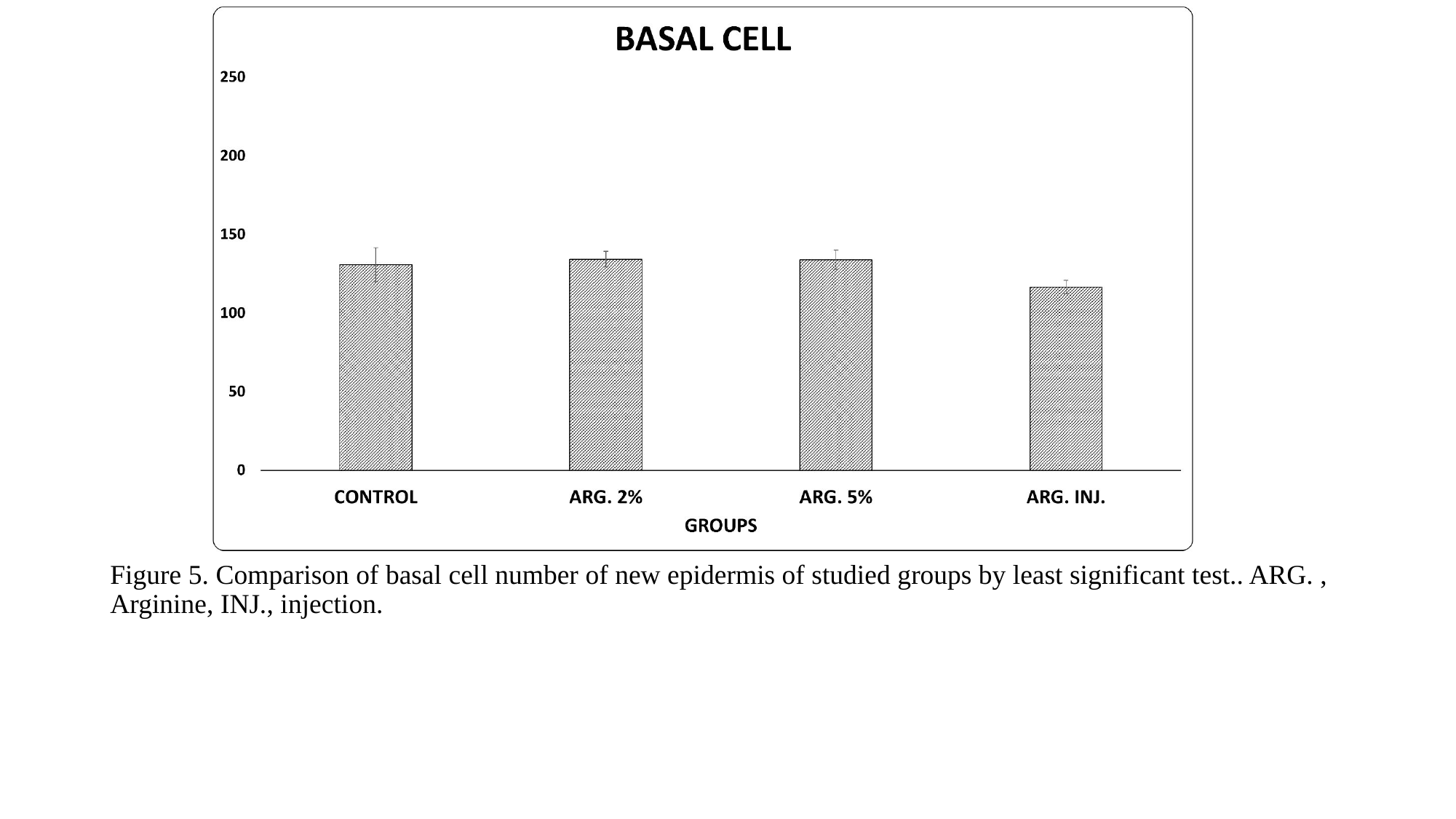

Figure 5. Comparison of basal cell number of new epidermis of studied groups by least significant test.. ARG. , Arginine, INJ., injection.

## Slide 6
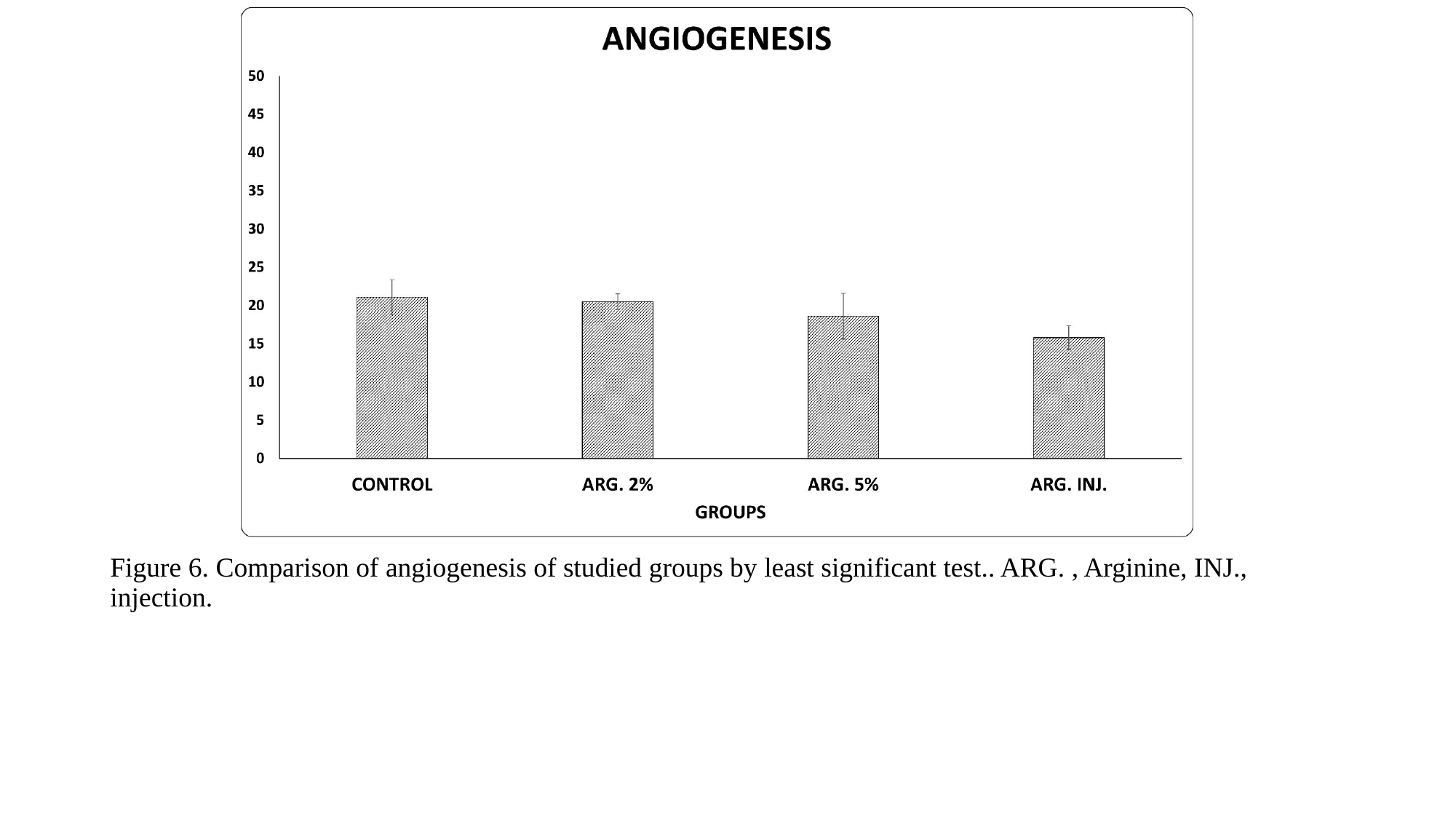

Figure 6. Comparison of angiogenesis of studied groups by least significant test.. ARG. , Arginine, INJ., injection.
